# Impulsive Lifestyle Counselling versus treatment as usual to reduce offending in people with co-occurring antisocial personality disorder and substance use disorder: a post hoc analysis

**DOI:** 10.1186/s12888-022-04025-8

**Published:** 2022-06-10

**Authors:** Morten Hesse, Adriana del Palacio-Gonzalez, Birgitte Thylstrup

**Affiliations:** Centre for Alcohol and Drug Research, Aarhus University, Bartholins Allé 10, 8000 Aarhus C, Denmark

**Keywords:** Antisocial personality disorders, Substance use disorders, Psychoeducation, Offending; Register-based study

## Abstract

**Objectives:**

To assess the impact of a short psychoeducation intervention for antisocial personality disorder on offending after randomization to treatment.

**Design:**

Multicentre, superiority, non-blinded randomized controlled trial. Random assignment was conducted in blocks of varying sizes at a central randomization centre. Participants were followed using national register data until 365 days after randomization, migration, or death, whichever occurred first.

**Setting:**

Thirteen outpatient uptake areas in Denmark.

**Participants:**

Patients with antisocial personality disorder in treatment for substance use disorders were randomized to treatment as usual (TAU, *n* = 80) or Impulsive Lifestyle Counselling (ILC, *n* = 96). A total of 165 patients could be linked to criminal records (TAU, *n* = 74; ILC, *n* = 91).

**Intervention:**

ILC is a brief psychoeducational program targeting antisocial behavior. The trial was conducted between January 2012 and June 2014.

**Outcomes:**

Number of criminal offences leading to convictions based on national registers.

**Results:**

The mean number of offences was 2.76 in the TAU group (95% Poisson confidence interval [CI] = 2.39, 3.16) and 1.87 in the ILC group (95% CI = 0.97, 1.43). Negative binomial regression was used to assess total number of convictions, as well as convictions for violent, property, driving under the influence, and drug-related crimes. In both adjusted and unadjusted analyses, random assignment to ILC was associated with a lower number of total offences (incidence rate risk ratio [IRR] = 0.43, *p* = .013; adjusted IRR = 0.45, *p* < .001) and convictions related to violence (IRR = 0.19, *p* = .001 adjusted IRR = 0.19, *p* = .007) and property offences (unadjusted IRR = 0.30, *p* = 0.003, adjusted IRR = 0.42, *p* = 0.010). Differences between conditions were not significant for driving under the influence (unadjusted IRR = 0.49, *p* = .370; adjusted IRR = 0.53, *p* = .417) or drug offences (unadjusted IRR = 1.06, *p* = .907; adjusted IRR = 0.55, *p* = .223).

**Conclusions:**

The ILC program shows promise in reducing offending behavior in people with comorbid substance use and antisocial personality disorder.

**Trial registration:**

ISRCTN registry, ISRCTN67266318, 15/10/2012.

## Background

Antisocial personality disorder (ASPD) is characterized by a pervasive disposition to disregard and disrupt the rights of others, frequent violation of the law, impulsivity, and hostility [1] and is strongly linked to having deviant peers and offending [2, 3]. While the disorder often improves with time, many people continue to experience related problems well into late adulthood [4, 5]. ASPD is often a complex condition and is highly comorbid with other disorders such as anxiety and mood disorders [6] and substance use disorders (SUDs) [7, 8]. Both ASPD [7] and SUDs are central to the externalizing spectrum of mental health problems [9, 10] and are more prevalent in men than in women in both general [11] and patient populations [12, 13].

Traditionally, people with ASPD have been described as ‘treatment rejecting’ [14], but much attention has been given to the importance of recognizing and managing conduct disorders and antisocial behavior in children, young people, and adults [15–17]. The few conducted ASPD treatment studies have generally had small samples; however, they have shown promising results, thus indicating that patients with a diagnosis of ASPD can be engaged in treatment and helped [18]. Despite a high co-occurrence of ASPD and other psychiatric disorders, such comorbidities have rarely been addressed by treatment studies. However, two recent studies examined intensive long-term treatment interventions for people with comorbid ASPD and borderline personality disorder. In one study, a mentalization-based intervention consisting of 140 individual and group sessions reduced symptoms related to antisocial behavior, including anger, hostility, and impulsivity [19]. In the other study, 30 patients with borderline personality disorder and antisocial behavior received Dialectical Behavior Therapy [20]. The authors reported a high rate of treatment completion and a significant reduction in a range of dysfunctional behaviors during treatment [20].

Although SUDs and ASPD frequently co-occur [8], studies of interventions targeting this comorbidity are also scarce. Some evidence indicates that antisocial traits are linked to lower levels of retention in psychosocial treatment, specifically among people with SUDs [21, 22], and that ASPD is associated with offending after discharge from SUD treatment [3, 23]. The lack of research on interventions targeting ASPD was highlighted in a recent Cochrane review that concluded that the few studies that exist do not support any psychological interventions for ASPD [24]. Another criticism raised in the Cochrane review was the absence of data on convictions after treatment in the existing literature. This is an important omission given that one of the criteria for ASPD is criminal behavior that could lead to convictions [1].

The Centre for Alcohol and Drug Research at Aarhus University, Denmark developed and tested an intervention addressing ASPD and SUD comorbidity, the Impulsive Lifestyle Counselling program (ILC) [25]. The program aims to raise awareness and support self-understanding of dysfunctional impulsive patterns of actions related to ASPD through psychoeducation [25]. The intervention was developed to address problems frequently associated with ASPD, such as impulsive actions, lack of self-control, and substance use [26, 27]. The intervention applies a non-judgmental and non-stigmatizing approach, while also acknowledging the difficulties associated with the patients’ behavior.

All six sessions in the ILC program focus on dysfunctional impulsive behavior related to ASPD in several ways. When patients are presented with the term ‘impulsive lifestyle’ in the first session, it is explained that this refers to impulsive actions that lead to substance use and conflicts with others, including the police. Further, patients are asked to consider four areas of impulsivity related to dysfunctional impulsive behavior (violations of others’ rights, rule violations, irresponsibility, and self-indulgence). In session two, patients are introduced to the Triggers-Actions-Consequences model that aims at supporting them in linking their behavior to immediate adverse consequences, such as loss of a partner and incarceration. This model, which is used throughout the workbook, allows the patients to recognize the link between impulsive patterns of action and negative outcomes in their everyday life. In a pragmatic multicentre clinical trial, the ILC was added on to treatment as usual (TAU) and tested in outpatient treatment for SUDs in Denmark [25].

The first paper from the trial focused on dropout from SUD treatment as the outcome and showed that the ILC condition was associated with a lower risk of dropout from treatment (hazard ratio = 0.063) [28]. It was also found that patients randomized to ILC reported more days abstinent at the three-month follow-up but not beyond [29]. No significant differences were found on self-reported aggression between the two treatment conditions, but aggression was reduced considerably at both the three- and nine-month follow-ups [29]. Further, in additional analyses, it was found that the participants who received TAU + ILC reported having received more help for antisocial behavior compared to participants who received TAU only [30]. Reporting having received more help for ASPD was in turn associated with better short-term outcomes, such as more days abstinent, lower risk for dropout from treatment, and higher treatment satisfaction.

Building upon the previous findings, the current study aimed to address the gap in the literature concerning whether interventions targeting patients with SUD and comorbid ASPD and impulsive behavior have the potential to decrease criminal behavior [24]. To this end, we followed patients who had participated in the ILC trial (TAU vs. TAU + ILC) up to one year after randomization through national register data.

## Methods

### Design and settings

A pragmatic randomized trial was conducted between January 2012 and June 2014 in 13 sites in Denmark [29]. Patients enrolled in free-of-charge community outpatient treatment for people with SUDs were approached by the clinical staff and assessed using the ASPD module from the Mini International Neuropsychiatric Interview, version 5 [31].

Inclusion criteria were being between 18–65 years old, seeking or currently receiving treatment for an SUD, meeting lifetime and last-year criteria for ASPD according to DSM-IV criteria, and being able to provide informed consent. The DSM-IV criteria for ASPD met by the patients were reviewed and explained individually to each patient before patients consented to participate in the study. Patients were excluded from the trial if they were participating in group therapy with another patient enrolled in the trial, had acute psychosis or severe brain damage, did not speak Danish, or had plans that would interfere with study participation over the next three months. All participating patients were informed that according to the MINI International Neuropsychiatric Interview [31], they met the criteria for ASPD.

### Participants

In total, 176 patients were randomized in the trial, and, of these, 165 (93%) could be identified in the registers (ILC: *n* = 91; 54%; TAU: *n* = 74, 44.9%). In all, 12.7% were women, the median age was 31.5, and the interquartile range was 25.5–38 years (Table 1). Nearly half (46.7%) were undergoing medication-assisted treatment (MAT) for opioid dependence. In the year prior to randomization, nearly half of both groups had committed a crime leading to conviction (ILC: *n* = 44, 48%; TAU: *n* = 36, 49%). There were minor differences in specific offending types between groups, but none approached statistical significance (ps > 0.10).

**Table 1:** Baseline statistics (*N* = 165)

	ILC (*N* = 91)	Percentage	TAU (*N* = 74)	Percentage	Statistic
**Gender**					
Female	12	12%	9	13%	χ^2^(1) = 0.04
Male	79	88%	65	87%	
**Age (median/inter-quartile rage)**	30.6/13.8		32.5/12.4		Kruskall-Wallis χ^2^(1) = 0.40
**Number of criteria for conduct disorder satisfied (median/inter-quartile rage)**	4(2)		4(2)		Kruskall-Wallis χ^2^(1) = 0.11
**Number of criteria for adult antisocial personality disorder satisfied (median/inter-quartile rage)**	5(2)		5(2)		Kruskall-Wallis χ^2^(1) = 0.19
**Medication-assisted treatment at baseline**					
No	49	54%	39	53%	χ^2^(1) = 0.02
Yes	42	46%	35	47%	
**Offending (past year)**					
Any	44	48%	36	49%	χ^2^(1) = 0.00
Violent offending	9	10%	10	14%	χ^2^(1) = 0.53
Property offending	28	31%	20	27%	χ^2^(1) = 0.28
Serious drug offending	20	22%	11	15%	χ^2^(1) = 1.35
**Psychiatric history (past 10 years, hospital-based contacts)**					
Mood or anxiety disorders (F32-F49X)	24	26%	28	38%	χ^2^(1) = 2.49
Personality disorders (F6X)	8	11%	12	13%	χ^2^(1) = 0.22
Any inpatient care	14	15%	20	27%	χ^2^(1) = 3.38
**Any alcohol-related problems (past 10 years)**^a^	30	33%	22	30%	χ^2^(1) = 0.20

^a^Hospital-based with ICD-10 F10 diagnoses or prescriptions for alcohol dependence, ATC N07BB

Psychiatric diagnoses from the Danish Psychiatric Central Research Register are summarized in Table 1. In total, 71 (43%) had at least one psychiatric diagnosis, and 34 (20.6%) had been in inpatient psychiatric care in the past 10 years. The most common diagnoses were mood or anxiety disorders (*n* = 52, 31.5%) followed by attention deficit/hyperactivity disorder (*n* = 11, 6.7%). No difference was found in the prevalence rate of diagnoses between the TAU group (*n* = 35, 47.3%) and the ILC group (36, 39.6%), χ^2^(1) = 1.00, *p* = 0.318.

### Randomization

Random assignment was conducted in blocks of varying sizes at the Centre for Alcohol and Drug Research. Blocks varied between four and six patients per block within each site. Clinicians were informed of the results of the randomization only after the baseline assessment had been completed.

### Interventions

#### Treatment as usual (TAU)

Patients in both treatment conditions had access to counselling and medication for drug use disorders in Denmark under the Act of Social Services § 101 and for alcohol use disorders under the Healthcare Act § 141. When patients were randomly assigned to the TAU condition, clinicians were explicitly asked to ensure that the patients got the highest possible level of care, based on mutual agreement between the counsellor and the patient. TAU always included access to MAT for patients with opioid use disorders, psychosocial support in the form of casework and counselling, as well as referral to residential rehabilitation if this was deemed relevant. At some clinics, a liaison psychiatrist saw patients onsite, whereas patients in other clinics were referred to an off-site psychiatrist for diagnosis and treatment of other psychiatric conditions, such as attention-deficit/hyperactivity disorder, anxiety, or depression.

#### Impulsive Lifestyle Counselling (ILC)

ILC is a six-session psychoeducational add-on module to usual care that focuses on raising awareness of maladaptive antisocial behaviors. In brief, the program is inspired by the Lifestyle Theory and the Lifestyle Change Program developed by Glenn D. Walters [32, 33]. The ILC program contains a number of elements from the Lifestyle Change Program, such as introducing the patient to the concepts of behavioral styles, actions, choices, and consequences in relation to crime and impulsive behaviors. However, in order to reach a wider group of people with comorbid SUDs outside of prison, the ILC program was adjusted to an individual format, and the lifestyle approach was labeled ‘impulsive lifestyle’ rather than ‘criminal lifestyle’, addressing impulsive behavior related to ASPD, including problems with substance use and conflicts with others. The six sessions in the ILC program cover topics related to antisocial behavior, including a simplified Triggers-Actions-Consequences model, streetwise pride, values that increase or decrease impulsive actions, how social contacts may support or challenge lifestyle changes, and a booster session in which the patient and the clinician summarize the sessions and discuss future work with lifestyle changes. The content of the ILC program has been elaborately discussed in previous papers [29, 34], with the workbook available as supplementary material for this article.

Clinicians who delivered the ILC sessions at the participating sites attended a one-and-a-half-day workshop, where they were introduced to ASPD and the ILC program and practiced using the workbook by role-playing the sessions. The clinicians were encouraged to try to complete the sessions in the ILC program on a weekly basis, except for the booster session, which was to be delayed for six weeks. Sessions were planned to last 45–60 min, and the median number of sessions completed was two [29].

### Registers and data linkage

Baseline data from the trial were linked with register data, including date of death, socio-demographic data, and criminal justice data on a secure Statistics Denmark server. Further, we combined data from different registers to obtain information on psychiatric and alcohol-related diagnoses registered over a 10-year period prior to randomization.

*The Central Criminal Register* was established in 1978 and contains information on offenses and offenders in criminal cases for use in criminal procedures. The information is updated on a regular basis by the police districts in Denmark and the departments of the National Commissioner of Police [35]. The register was used in this study to obtain information on convictions up to one year after randomization.

*The Danish Psychiatric Central Research Register* contains diagnoses given by a medical doctor based on ICD-10 codes, as well as dates of treatment onset and termination [36]. While validation studies have been limited to specific diagnoses, the register is almost complete for hospital-based care, and thus most patients with moderate to severe mental health problems are likely to be included [36].

*The National Patient Register* was used to obtain alcohol-related diagnoses and covers all hospital contacts for somatic conditions [37].

*The Danish Prescription Register* was used to obtain information about prescription drugs received for alcohol use disorders [38]. This register contains information about all prescriptions filled by residents in Denmark, and each record contains the ATC code for the drug, the date of prescription filling, and the patient’s individual identification number.

### Outcomes

The primary outcome was the total number of crimes committed during the first year after study randomization leading to a conviction (i.e., not a warning or charges dropped). Secondary outcomes included number of specific offences that could be directly linked to antisocial behavior: property offences, violent offences, drug-related offences (excluding simple possession of drugs for own use), and driving under the influence of alcohol and drugs (DUI). In order to avoid small cells in the analyses, violent offences included sexual offences and weapons offences, as they both involve aggressive behavior towards others [39].

For both the primary and secondary outcomes, we considered only the first year after randomization as the observation period. This timeframe allowed enough time for the patients to commit an offence, while at the same time being able to observe treatment effects that may otherwise lose strength over a longer period due to external factors, such as life events or relapse to severe substance use [40]. The date of crime was the date at which the police believed that the criminal activity was initiated according to the recorded charge(s).

### Control variables

In all analyses, models adjusted for age, gender, and MAT at baseline, similar to previous reports from this trial [29], as well as previous offending of the same type in the year prior to randomization. All of these variables could potentially influence offending behavior. Crime rates differ by gender [41] and decline with age [5], and both male gender and lower age are associated with crime within samples of patients treated for substance use disorders [42].

#### Demographic and clinical variables

To better characterize our sample, we assessed the presence of severe psychiatric illness, mood or anxiety disorder, attention deficit/hyperactivity disorder, substance induced psychoses, or alcohol- related problems, all within a time frame of 10 years prior to randomization (3650 days).

Severe mental illness was defined as the presence of a schizophrenia spectrum disorder (F2X) or a bipolar disorder (F30-F319) diagnosis in the Danish Psychiatric Central Research Register. Mood or anxiety disorder was defined as the presence of either a unipolar mood disorder (F32-F99X) or an anxiety disorder (F4X). A personality disorder was defined as the presence of a personality disorder regardless of type (F6X), and hyperkinetic disorder was defined as the presence of an F900X diagnosis.

We defined alcohol-related problems as any hospital contact, inpatient or outpatient, involving an alcohol use disorder diagnosis (ICD-10 code F10X) identified in the National Patient Register or the Psychiatric Research Register, or the filling of a prescription for a drug used in the treatment of alcohol dependence (ATC code N07BB) identified in the Danish Prescription Register.

### Statistical analyses

Since the outcomes were count variables, we first explored models appropriate for this type of variable (i.e., Poisson, negative binomial, and zero-inflated models). To select the most parsimonious model, we relied on the Bayesian Information Criterion (BIC, [43]). The BIC takes on lower values as the model becomes more parsimonious, taking both model fit and model complexity into consideration.

Analyses for the best model were conducted with ILC randomization status as the variable of interest. In additional steps, we included the control variables listed above. According to the BIC, the best-fitting model was simple negative binomial regression for all outcomes in this study.

## Results

During the follow-up, the patients committed 312 offences leading to convictions, corresponding to a mean of 1.89 offences per person (standard deviation = 6.26). The mean number of offences in the TAU group was 2.76 (95% Poisson confidence interval [CI] = 2.39, 3.16), and, in the ILC group, the mean number of offences was 1.87 (95% CI = 0.97, 1.43).

Table 2 shows the proportion of patients who offended zero, one to two, or three or more times in the year after randomization, stratified by treatment assignment. A more fine-grained description by number of offences would violate rules against downloading micro-data from the Statistics Denmark server and therefore cannot be reported. Marginally more patients randomized to the ILC condition were crime-free (63.7%) compared with the TAU condition (53.3%), slightly more had offended one or two times (26.4% vs. 20%), and fewer had offended three or more times (9.9% vs. 26.7%). Note that these data are provided only for descriptive purposes and not in relation to hypothesis testing.

**Table 2:** Proportion with convictions by treatment assignment (*n* = 165)

**Number of convictions in year after randomization**	**Treatment as usual (*n* = 74)**	Percentage	**Treatment as usual plus Impulsive Lifestyle Counselling (*n* = 91)**	Percentage
None	39	52.7%	58	63.7%
One or two	15	20.3%	24	26.4%
Three or more	20	27.0%	9	9.9%

### Prediction of criminal offences: Count regression models

The results of the count variable regressions are summarized in Table 3. Randomization to the ILC condition was associated with a lower number of total convictions, both in the unadjusted (IRR = 0.43, *p* = 0.013) and adjusted (adjusted IRR = 0.45, *p* = 0.008) models. Regarding specific types of offences, randomization to the ILC condition was associated with a lower number of offences related to violence (unadjusted IRR = 0.19, *p* = 0.007, adjusted IRR = 0.19, *p* = 0.007) and property offences (unadjusted IRR = 0.30, *p* = 0.003, adjusted IRR = 0.41, *p* = 0.010). There were no significant differences between the treatment groups with regard to DUI and drug offences (DUI: unadjusted IRR = 0.49, *p* = 0.371, adjusted IRR = 0.62, *p* = 0.548; drug offences: unadjusted IRR = 1.06, *p* = 0.905; adjusted IRR = 0.55, *p* = 0.223).

**Table 3:** Effects of the Impulsive Lifestyle Counselling add-on on all criminal justice outcomes (negative binomial regression)

	**Unadjusted IRR**^a^	**95% CI**^b^	***P*-value**	**Adjusted** **IRR**	**95% CI**	***P*-value**
Total convictions	0.43	0.22, 0.83	.013	0.43	0.24 – 0.78	< .001
Property crimes	0.30	0.14, 0.66	.003	0.40	0.20– 0.79	.009
Violent crimes	0.19	0.06, 0.64	.007	0.18	0.05 – 0.62	.006
Drug crimes	1.06	0.38, 2.97	.905	0.59	0.23 – 1.51	.180
Driving under the influence of alcohol and drugs	0.49	0.10, 2.34	.371	0.62	0.13 – 2.91	.521

^a^Incident Rate Ratio

^b^Confidence interval

### Other variables associated with offending

The number of offences before randomization to the study was associated with the total number of offences after randomization (IRR = 1.28; 95% CI = 1.14, 1.43). Similarly, pre-randomization number of property offences was associated with post-randomization number of property offences (IRR = 1.51; 95% CI = 1.23,1.85). Pre-randomization number of drug offences was associated with later drug offences (IRR = 2.37; 95% CI = 1.43, 3.95), and a history of alcohol-related problems was associated with a lower risk of drug offences (IRR = 0.26; 95% CI = 0.08, 0.86). No variables were significantly associated with number of DUI offences after randomization.

## Discussion

To our knowledge, this is the first study to assess whether an intervention targeting people with comorbid ASPD and SUD would reduce criminal offending [24]. The results are promising, as patients randomly assigned to TAU with the add-on of the ILC program had fewer criminal offences than those assigned to TAU in the year following randomization, especially violent offences and property offences. However, no significant difference was observed between the two treatment conditions with regard to drug-related and DUI offences. The reasons for the non-significant differences between the conditions with regard to these two offending categories probably differ. It is likely that drug offences are less related to impulsive behavior and decrease in substance use [44, 45] since serious drug offences require some planning and coordination. While DUI offences were very rare in our sample, and only 4% were convicted of DUI in the year following randomization, other studies that have reported high rates of acts of violence [46–48] and higher rates of offences related to DUI [49] among people with ASPD and antisocial personality disorder traits.

We can only speculate about possible mechanisms of action that have contributed to the results in the present study. The components of the ILC program focus on inviting the patient to consider whether aspects of ASPD related to what is termed “an impulsive lifestyle” in the ILC program match his or her own experience and self-image and whether it makes sense to him or her to change these behaviors. Further, the components of the program focus on the individual patient’s own experiences of costs and benefits linked to his or her behavior. The patient is asked to evaluate specific situations with adverse outcomes in terms of what could be the link between triggers, behaviors, and these outcomes, thereby increasing his or her awareness of the link between behaviors associated with ASPD and unwanted consequences. If successful, the ILC program increases this awareness and supports the individual patient in changing or controlling maladaptive antisocial behaviors and impulses [50]. As such, the aim of the program is to support an improved awareness in patients with ASPD that could be expressed in real-life changes, such as increasing engagement in treatment and desisting from use of substances and offending behavior. Thus, while a brief psycho-education program may not change all aspects of ASPD, our earlier findings showed that patients who had received ILC sessions experienced that they received more help for ASPD [30], had lower risk of drop out [28], and reported more abstinent days [29].

Given that the link between mechanisms of action and outcomes was not the focus in the present study, it is not possible for us to report on the extent to which changes in ASPD and impulsive behaviors may have driven the effects on crime and offending behavior. However, it may be that the ILC program had an indirect effect on offending behavior by decreasing antisocial and impulsive behavior and by increasing self-control, as suggested by Wojciechowski [26]. However, previous research suggests that, even during active treatment, only small changes in impulsivity occur among SUD patients [51], while treatment of impulsive behaviors among patients without SUD appears more promising [52]. Future research on interventions targeting ASPD and other comorbid conditions, such as the ILC, would benefit from studying the link between mechanisms of action and outcomes.

It is important to note that our sample was heterogeneous in terms of substances used. Certain types of drugs may directly increase the risk of crime due to pharmacological effects, may cause patients to engage in crime as part of the struggle to obtain drugs or money to pay for drugs, or may involve patients in environments where illegal behaviors, such as violence or property crime, are part of the social practices. Known as Goldstein’s tripartite model, this perspective suggests that drug use may increase other risks [53]. Our findings lend indirect support for this model [54], although the patients in the two conditions in the ILC trial reported a quite similar use of drugs.

Two control variables predicted criminal offending after randomization: a history of previous offending in the year leading up to randomization predicted a higher risk of offending, and MAT predicted a lower risk of offending. Previous offending is robustly associated with new offending in the scientific literature [55]. MAT was available to patients who were opioid dependent in our study sample. Therefore, because MAT was highly confounded with substance type in this study, the findings cannot be interpreted as an indication of whether receipt of MAT is associated with lower risk of offending. However, future studies should involve and compare patient groups with and without opioid use disorders [56].

The patients in this study had a high prevalence of comorbid mental health conditions, with nearly half diagnosed in a psychiatric setting in the 10 years prior to randomization. These findings are not surprising, as we have found similar rates of psychiatric history in other samples of patients in SUD treatment in Denmark [57] and comorbidity with other psychiatric diagnoses is common among patients with comorbid ASPD and SUD [58]. While it has been shown that ASPD is linked to committing more criminal offences, it is unclear whether comorbidity with other mental health problems, such as borderline personality disorder, may increase this risk [48]. In the present study, we did not find that the presence of other comorbid psychiatric conditions had a significant impact on offending, or that the results of the ILC program were affected by having a psychiatric comorbidity.

The greatest strength of this study is the longitudinal before-after design, which offers a unique opportunity to study reported criminal offences in the year following study intake. However, there were several limitations in our study. First, the sample size was not large enough to assess the impact of specific psychiatric comorbidities. Similarly, the small number of women in this study precluded robust analyses of the degree to which the results can be generalized to women with comorbid ASPD and SUD. Only the experimental group received a bona fide add-on to TAU, and there is evidence that TAU may not be an optimal comparison for an active treatment [59]. Further, as with any register-based study, we were not able to provide direct quality control over the process of data collection and were only able to include offences recorded by the police. It is highly likely that other offences were committed in the same time period but were not recorded (e.g., drug sales). Thus, the study provided a conservative estimate of offences committed during the study period, but, as this was the case for the ILC and TAU conditions, this limitation was not likely to have had any impact on the difference in outcomes between the two conditions. Lastly, we did not directly assess impulsivity as an outcome of our study.

## Conclusions

The short-term ILC program targeting antisocial behavior and criminal offending has the potential to reduce offending behavior among people with antisocial personality disorder.

## Data Availability

The datasets generated and/or analysed during the current study are not publicly available due to data protection legislation, but are available from the corresponding author on reasonable request.

## Abbreviations

ASPD: Antisocial personality disorder
ILC: Impulsive Lifestyle Counselling
IRR: Incident Rate Ratio
SUD: Substance use disorder
TAU: Treatment as usual
